# Comparative Transcriptome Sequencing Analysis Revealed Key Pathways and Hub Genes Related to Gill Raker Development in Silver Carp (*Hypophthalmichthys molitrix*)

**DOI:** 10.3390/biology14121797

**Published:** 2025-12-17

**Authors:** Xiaohui Li, Ziyang Geng, Cui Feng, Hongwei Liang

**Affiliations:** 1 Yangtze River Fisheries Research Institute, Chinese Academy of Fishery Sciences, Wuhan 430223, China; lixiaohui@yfi.ac.cn (X.L.); gzyy1021@163.com (Z.G.); fc047142@163.com (C.F.); 2 College of Fisheries and Life Science, Shanghai Ocean University, Shanghai 201306, China; 3 Key Laboratory of Aquatic Genomics, Ministry of Agriculture and Rural Affairs, Chinese Academy of Fishery Sciences, Beijing 100141, China

**Keywords:** *Hypophthalmichthys molitrix*, gill raker, development, molecular changes, transcriptome

## Abstract

The silver carp (*Hypophthalmichthys molitrix*) is a filter-feeding fish species, characterized by significant morphological transformations in its filter-feeding apparatus, particularly the gill rakers, which are closely associated with dietary changes throughout its development. Despite the importance of these morphological innovations, the molecular mechanisms driving these changes remain largely unexplored. To investigate this, we employed an integrative approach combining scanning electron microscopy (SEM) and comparative transcriptomics to examine the gill rakers at five critical developmental stages (6, 15, 30, 45, and 60 days post-hatching, dph). SEM analysis revealed a structural evolution from sparse, bump-like protrusions to a dense, interlocking mesh. Simultaneously, transcriptomic analysis identified 10,184 differentially expressed genes (DEGs), which showed significant enrichment in pathways such as Focal Adhesion, ECM-Receptor Interaction, and the PI3K-Akt Signaling Pathway. Gene Set Enrichment Analysis (GSEA) indicated a coordinated upregulation of collagen and integrin gene families during the early developmental transition (6 vs. 15 dph), highlighting their crucial role in the formation of the sieve structure. This study reveals the molecular mechanisms of gill raker development in silver carp, providing initial insights into genetic regulation of morphology for ecological adaptation. The findings connect developmental biology, evolutionary biology, and ecology.

## Simple Summary

The silver carp (*Hypophthalmichthys molitrix*) is a filter-feeding fish that utilizes its gills to extract plankton from water, thereby contributing to the regulation of algal growth and the maintenance of ecological balance. The gills, particularly the gill rakers, are vital for feeding, and their development is closely associated with dietary changes. However, the molecular alterations involved in gill development in this species remain unexplored. This study examines the gill structure of silver carp at 6, 15, 30, 45, and 60 days post-hatching (dph) and elucidates the molecular mechanisms underlying gill development. It provides preliminary insights into the genetic regulation of morphological construction in response to natural selection, thereby facilitating precise adaptation to ecological environments. The findings establish a crucial connection between developmental biology, evolutionary biology, and ecology.

## 1. Introduction

The fish gill is a multifunctional organ that, beyond its role in gas exchange, is critically involved in feeding, particularly among filter-feeding species where this function is of utmost importance [1,2]. This feeding mechanism is primarily enabled by a specialized morphological structure known as the gill raker, which function as a selective sieving apparatus [3]. As water carrying food particles flows through the oral cavity into the gill chamber, the intricately and densely arranged gill rakers form the initial physical barrier [4,5]. They filter and retain a variety of food particles, including phytoplankton and zooplankton, preventing their escape through the gill slits and directing them toward the oropharynx for ingestion, after which the particles are conveyed to the esophagus [6,7]. Consequently, the morphology of the gill rakers—characterized by their length, density, and complexity—directly influences the feeding efficiency and dietary selectivity of fish [8,9,10], serving as a key adaptive organ that connects the fish to its trophic environment. It is precisely the variation in gill raker morphology that drives trophic diversification and niche differentiation among various fish species [11].

The silver carp (*Hypophthalmichthys molitrix*) is a representative filter-feeding fish species that plays a crucial role in the riverine and lacustrine ecosystems of China [12]. Its distinctive feeding mechanism is entirely dependent on highly specialized gill rakers, which are elongated, densely arranged, and possess interlocking lateral branches that create an intricate mesh-like filtration screen [4,13]. As water traverses the oral cavity, this screen effectively filters out the majority of phytoplankton and organic detritus. This highly efficient filter-feeding strategy allows the silver carp to consume substantial quantities of primary productivity in aquatic environments, thereby fulfilling an indispensable ecological function in controlling algal blooms and mitigating eutrophication [14]. Owing to its remarkable filter-feeding capability and significant potential for algal bloom management, the silver carp has been introduced to numerous countries globally as a vital biological tool for enhancing water quality and managing eutrophication [15].

Throughout its ontogeny from larval to adult stages, the silver carp experiences a pivotal dietary transition from active ingestion to passive filter-feeding, a process intricately aligned with the morphological development of its gill rakers. During the initial developmental phase (total length 7–14 mm), the larvae predominantly consume small zooplankton, while their gill rakers are characterized by a limited number of sparse, tubercle-like protrusions lacking filtering capabilities [16]. As the organism progresses into the dietary transition phase (total length 15–29 mm), there is a rapid increase in both the number and length of the gill rakers, which gradually evolve to exhibit complex lateral projections (side spines) [16]. Ultimately, in the filter-feeding stage (total length > 30 mm), these side spines interlock to form a dense, interwoven, and highly efficient mesh-like sieve structure [16]. This structural metamorphosis facilitates a complete transition in the silver carp’s feeding strategy—from visually guided active predation to passive filter-feeding, which relies on the gill rakers to capture microscopic phytoplankton—thereby solidifying its ecological niche [17,18].

The relationship between gill raker development and dietary transition in silver carp has been preliminarily established; however, the molecular mechanisms underlying the formation of this intricate structure remain largely undefined. The transformation from sparse, tubercle-like protrusions to a complex, mesh-like filtration apparatus is likely regulated by specific genetic programs and molecular pathways [3]. Although transcriptomics has emerged as a powerful tool for studying gene expression and has been utilized in various fish species to investigate gill adaptations to environmental conditions [19,20,21,22], the specific molecular programs driving gill raker development in silver carp, particularly the dynamic gene expression patterns and core regulatory networks, have not been systematically characterized. To elucidate the molecular basis of gill raker development in silver carp, this study employed transcriptome sequencing to conduct a systematic analysis of gill raker across key developmental stages, ranging from the initial swallowing phase to the filter-feeding stage. By constructing gene expression profiles, we aimed to identify differentially expressed genes critical to this morphogenetic process and uncover their regulatory pathways, thereby clarifying the molecular mechanisms behind the formation of the unique filter-feeding apparatus in silver carp, thereby offering crucial insights into the comprehensive “gene-form-function-ecology” continuum.

## 2. Materials and Methods

### 2.1. Fish Preparation and Experimental Design

The silver carp utilized in this study were sourced from the seed multiplication farm of the Yangtze River Fisheries Research Institute, located in Jingzhou City, Hubei Province, China. In May, when water temperatures range from 24 °C to 26 °C [23], mature female and male broodstock are induced to spawn through the administration of ovulation-inducing hormones (LRH-A2 10 µg/kg) and subsequently transferred to spawning pools to facilitate successful reproduction. Fertilized eggs are collected from these spawning pools and incubated in hatching tanks. Upon hatching, once the fry are capable of sustained horizontal swimming, they are nourished with egg yolk until 6 dph, at which point they achieve a total length of 6–7 mm. The larvae are then relocated to outdoor concrete tanks measuring 3 m in width, 6 m in length, and 2 m in depth, where they are reared until 60 dph. One week prior to the introduction of the larvae, an Algal-Bacterial Polypeptide (Linckrich, Shenzhen, China) was applied to enhance the plankton biomass in the water, which comprised both phytoplankton (e.g., unicellular algae) and zooplankton (e.g., rotifers and cladocerans) [24], thereby providing an adequate food source for the juvenile fish. During the entire rearing period, critical environmental parameters were systematically monitored and regulated to minimize external influences on developmental processes. The water temperature was naturally sustained within the optimal range for silver carp growth (24–28 °C) due to prevailing seasonal conditions, and the photoperiod adhered to the natural light-dark cycle characteristic of the region.

### 2.2. Sample Collection

Owing to the diminutive size of the gill rakers in juvenile silver carp, a comprehensive collection of specimens was undertaken to ensure an adequate sample size for subsequent experimental analyses. Specifically, a total of 120 larvae were collected at both 6 and 15 dph, divided into three groups of 40. For juveniles, 60 were collected at both 30 and 45 dph, divided into three groups of 20. Additionally, 21 juveniles were collected at 60 dph, divided into three groups of 7. All fish were anesthetized using 50 mg/L of MS-222 (Sigma, St. Louis, MO, USA) prior to the measurement of their total length and body weight. The anesthetized specimens were subsequently positioned under a stereomicroscope (SOPTOP SZM, Yuyao, China). Utilizing fine-tipped forceps, the operculum was meticulously removed to reveal and extract the complete gill apparatus. A subset of the samples was preserved in 2.5% glutaraldehyde (Servicebio, Wuhan, China) for subsequent scanning electron microscopy (SEM) analysis. Another subset underwent processing involving the removal of the gill filaments; the remaining gill raker tissues were immediately flash-frozen in liquid nitrogen and stored at −80 °C for future transcriptomic analysis. All animal experiments were performed in compliance with the Guiding Principles for the Care and Use of Laboratory Animals, as approved by the Animal Experimental Ethical Inspection of the Laboratory Animal Centre, Yangtze River Fisheries Research Institute, Chinese Academy of Fishery Sciences (ID number: 2020-LXH-01).

### 2.3. Scanning Electron Microscopy Analysis

The gill raker morphology of silver carp was examined using scanning electron microscopy (SEM) [3]. The procedures for fixation, sample processing, and microscopic observation strictly followed the previously described methodology [25]. Gill tissues fixed in 2.5% glutaraldehyde for over 24 h were retrieved and rinsed three times with phosphate buffer. Following this, the samples were post-fixed with 1% osmium tetroxide at 4 °C for 1 h and then washed with phosphate-buffered saline (PBS). Subsequently, the samples were dehydrated through a graded ethanol series (50% to 100%), with the medium then replaced by isoamyl acetate for conventional drying. Upon completion of these preparation steps, the specimens were ion-coated under vacuum and imaged using a scanning electron microscope (Hitachi, SU8100, Tokyo, Japan).

### 2.4. Total RNA Isolation and Sequencing

Total RNA was extracted from the gill raker samples using the TRIzol reagent (Invitrogen, Foster City, CA, USA) following to the manufacturer’s protocol. The variation in the number of individuals per experimental group across different developmental stages is primarily due to differences in gill raker size and the corresponding total RNA yield. At the early stages (6 and 15 dph), the gill raker tissue is extremely minute, requiring the pooling of 40 individuals per experimental group to obtain the minimum amount of high-quality RNA (2 μg) necessary for robust transcriptome sequencing. As the fish grow, the gill rakers develop significantly, allowing for a gradual reduction in the number of individuals required per experimental group—20 individuals at 30 and 45 dph, and only 7 individuals at 60 dph—to achieve the same RNA input quantity. The RNA quality and integrity were estimated using electrophoresis through a 1% agarose gel and using a Bioanalyzer 2100 (Agilent Technologies, Foster City, CA, USA). The RNA concentration was further evaluated using a NanoDrop instrument (Thermo Scientific, Waltham, MA, USA).

A total of 15 RNA samples (2 μg each) form 6 dph–60 dph gill raker, were used for the cDNA library construction and sequencing. The cDNA libraries were constructed using a TruSeq™ RNA Sample Preparation Kit (Illumina, San Diego, CA, USA), and the libraries were sequenced using the Illumina NovaSeq 5000 system and a double-ended read length of 150 bp was generated by Shanghai Paisennuo Biotechnology Co., Ltd. (Shanghai, China) All the data are available at the CNCB GSA database (GSA numbers: CRA033098).

### 2.5. Analysis of RNA-Seq Data

The analysis of RNA sequencing (RNA-seq) data was carried out, and the specific details are as follows. The quality control of Fastq output files was assessed using FASTQC (v0.11.5) [26]. Adapter sequences were removed using Trimgalore (v0.4.3) [27]. The quality-controlled reads were aligned to the reference genome of silver carp (GCA_041475455.1) using HISAT2 (v2.1.0). Raw counts were generated using HTSeq (v0.6.1) [28]. Normalization of gene expression and differential expression genes (DEGs) analysis were performed using DESeq2 (v1.30.1) [29]. Pairwise comparisons were performed between the following groups: 6 dph vs. 15 dph, 6 dph vs. 30 dph, 6 dph vs. 60 dph, 15 dph vs. 30 dph, and 30 dph vs. 60 dph. A fold change (FC) > 2.0 and an adjusted *p*-value < 0.05 threshold (false discovery rate, FDR) were used as criteria for identifying DEGs. Hierarchical clustering of DEGs was performed using the heatmap package in R (version 3.8.2) [30]. Gene ontology (GO) enrichment analysis for DEGs was conducted using the Bioconductor R package clusterProfiler (v3.18.1) [31] with a significance threshold of *p* < 0.05 [31]. Co-expression network and hub gene analysis: To identify key regulatory genes within the significant clusters derived from GO analysis, co-expression networks were constructed for each cluster based on Pearson correlation (|r| > 0.95). Hub genes were identified from these networks using the Maximal Clique Centrality (MCC) algorithm within the CytoHubba plugin of Cytoscape (v3.9.1), which identifies nodes central to highly interconnected subnetworks. Kyoto Encyclopedia of Genes and Genomes (KEGG) pathway enrichment analysis was subsequently performed on the DEGs using clusterProfiler (v3.18.1) [31] with *p* < 0.05 [31]. GSEA analyzes gene expression by grouping genes into functional or pathway-based sets, offering a broader view of expression patterns. We utilized GSEA software (v4.0.3) [32] (https://www.omicshare.com/ (accessed on 20 October 2024) and MsigDB database (v2023.1) [33] to perform pathway enrichment analysis for the 6 dph vs. 15 dph comparison, focusing on the early developmental transition. Enrichment scores and *p*-values were calculated using signal-to-noise normalization on the gene expression matrix and ranked genes with default settings.

### 2.6. Real-Time Quantitative PCR (RT-qPCR) Analysis

The total RNA extracted from each group was reverse-transcribed using the EasyScript^®^ One-Step gDNA Removal and cDNA Synthesis SuperMix (TransGen, Beijing, China) according to the manufacturer’s instructions. The cDNA was then subjected to RT-qPCR using the ABI 7500 Real-Time PCR System (Applied Biosystems, Foster City, CA, USA) with gene-specific primers, and 40S ribosomal RNA was used as an internal control. The primer sequences are shown in Table S1. The gene expression levels were calculated using the comparative Ct method (2−ΔΔCt) with QuantStudio™ Real-Time PCR Software, version 1.2 [34].

### 2.7. Statistical Analysis

The total length and weight data of the fish are presented as the mean ± standard error (M ± SE). The rest data were analyzed using one-way analysis of variance (ANOVA) with Tukey’s post hoc test at a 95% confidence level [35], performed using GraphPad Prism software (version 9.0). The results of RT-qPCR were evaluated using Student’s *t*-test. A *p*-value of less than 0.05 was considered statistically significant.

## 3. Results

### 3.1. Morphological Changes in Gill Rakers of Silver Carp Across Developmental Stages

**Figure 1:**
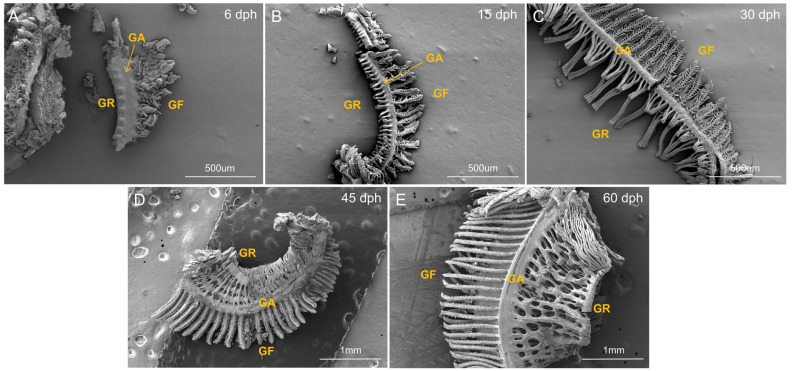
Scanning electron micrographs showing the morphology of gill rakers in silver carp at different developmental stages. (**A**) 6 dph; (**B**) 15 dph; (**C**) 30 dph; (**D**) 45 dph; (**E**) 60 dph. GR, gill raker; GA, gill arch; GF, gill filament. Scale bars are indicated in each panel.

In order to ensure that the gill raker samples utilized for transcriptome analysis accurately reflect the developmental progression of gill rakers in silver carp, morphological assessments were performed on specimens from five distinct developmental stages (6, 15, 30, 45, and 60 dph). These assessments encompassed measurements of total body length and body weight (Table 1), alongside histological examinations of gill raker tissues (Figure 1).

In the initial developmental phase (6–15 dph), the larvae exhibited a total length ranging from 7 to 14 mm. At this stage, the gill rakers were observed as a limited number of short, tubercle-like protrusions, which were sparsely distributed and not yet operational for filter feeding (Figure 1A,B). During this period, the larvae predominantly depended on the absorption of the yolk sac and the ingestion of small zooplankton, indicative of an active predation stage [16]. During the juvenile stage (15–45 dph) when the organism reaches a total length of 15–30 mm, the gill rakers experience significant morphological changes. There is a marked increase in their number and a rapid elongation, accompanied by a structural transformation from tubercle-like protrusions to forms characterized by multiple lateral projections, or side spines. These adjacent gill rakers start to interlock through the side spines, initiating the formation of a filtering screen that covers the branchial cavity (Figure 1C,D). During this phase, the feeding behavior shifted from active predation to passive filter feeding [16]. By the late juvenile stage, at 60 dph, the total length surpassed 30 mm. At this stage, the development of the gill rakers and their lateral spines was largely complete, forming a dense, interlocking, mesh-like structure (Figure 1E). Consequently, the gill rakers were fully developed and functionally mature, acting as an efficient sieve for retaining larger particles, thereby signifying the silver carp’s definitive transition to a filter-feeding lifestyle [16].

### 3.2. The Quality Assessment of Transcriptome Data

To elucidate the molecular mechanisms underlying the development of the gill raker in silver carp, total RNA was extracted and subjected to RNA sequencing analysis from three biological replicates across five developmental stages (6 dph, 15 dph, 30 dph, 45 dph, and 60 dph). The quality metrics for clean reads, with Q20 and Q30 values exceeding 97.8% and 95.8%, respectively, demonstrate that the majority of raw reads generated by high-throughput sequencing met the quality standards (Table S2). Post-quality filtering, the paired clean reads were aligned to the silver carp genome. The percentages of total mapped reads (ranging from 90.71% to 93.05%), reads mapped to genes (86.02% to 89.98%), reads mapped to intergenic regions (10.67% to 13.98%), and reads mapped to exons (93.88% to 95.85%) were consistent across the 15 samples (Table S2). The high Q20/Q30 scores, coupled with the high mapping rate, ensured the accuracy and reliability of the gene expression matrix, providing a robust foundation for subsequent differential expression and functional enrichment analyses.

### 3.3. Differential Gene Expression Analysis During Gill Raker Development

**Figure 2:**
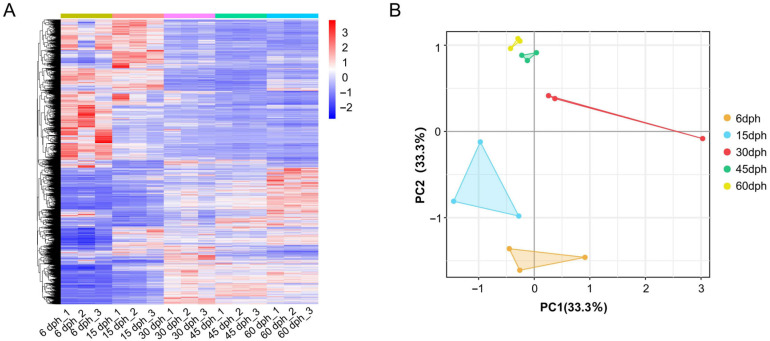
Analysis of gene expression profiles in the gill raker of silver carp. (**A**) Heatmap of normalized expression values (Z-score) for all detected genes across five developmental stages: 6, 15, 30, 45, and 60 dph. Each column represents a biological replicate. The color scale from blue to red indicates low to high relative expression. (**B**) Principal component analysis conducted on 15 gill raker samples.

A total of 25,387 genes were identified from the 15 gill raker samples of silver carp, with their relative expression levels across developmental stages depicted in Figure 2A and detailed in Table S3. Principal component analysis (PCA) demonstrated strong reproducibility among biological replicates (Figure 2B). The 30 dph replication demonstrated greater variability; however, its sequencing quality was consistent with that of other samples, and no global expression bias was detected. This observed variation can be attributed to inherent biological asynchrony occurring during rapid developmental stages. Furthermore, the PCA graph demonstrates a high degree of consistency between the 45 dph and 60 dph samples. When considered alongside the gill raker morphology depicted in Figure 1D,E, it is evident that the sieve-like structure of the gill rakers at 45 dph is already well developed and closely resembles that observed at 60 dph. To facilitate more efficient analyses, we have elected to retain only the 60 dph samples for further investigation.

**Figure 3:**
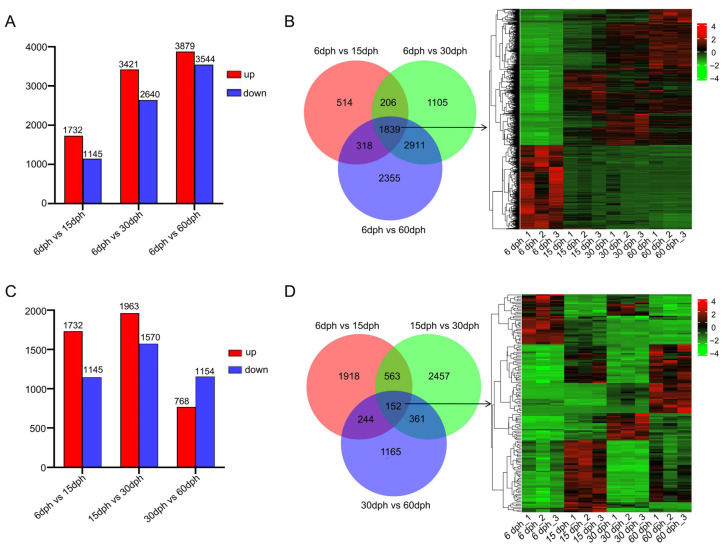
Examination of DEGs in the gill rakers of silver carp across various developmental stages. (**A**) The quantity of DEGs identified relative to the 6 dph group. (**B**) A Venn diagram illustrating the overlapping DEGs among the three comparison groups in (**A**), accompanied by a heatmap depicting the expression patterns of these shared DEGs. (**C**) The number of DEGs identified at each developmental stage in comparison to the preceding time point. (**D**) A Venn diagram demonstrating the overlapping DEGs among the three comparison groups in (**C**), along with a heatmap representing the expression patterns of these shared DEGs.

Differentially expressed genes (DEGs) were analyzed using two distinct methodologies, with one approach designating the 6 dph group as the control (Table S4). As illustrated in Figure 3A, relative to the 6 dph group, 2877 DEGs were identified in the 15 dph group, comprising 1732 up-regulated and 1145 down-regulated genes. In the 30 dph group, a total of 6061 DEGs were detected, including 3421 up-regulated and 2640 down-regulated genes. The 60 dph group exhibited 7423 DEGs, with 3879 up-regulated and 3544 down-regulated. These findings indicate a temporal increase in the number of differentially expressed genes. A Venn diagram revealed that 1839 DEGs were common to all three comparison groups (Figure 3B, Table S5). These genes underwent clustering analysis based on their expression levels, as depicted in a heatmap (Figure 3B). The 1839 DEGs were broadly categorized into two major groups: one demonstrating a continuous up-regulation trend over developmental time, and the other exhibiting a continuous down-regulation trend. The persistent up- and down-regulation of these genes was maintained throughout the entire developmental process.

The alternative method for analyzing DEGs involved a comparative analysis of each subsequent time point with its preceding stage (Table S4). Figure 3C presents the outcomes of these sequential comparisons across various developmental stages. The analysis revealed that, relative to the 6 dph group, 2877 DEGs were identified in the 15 dph group, comprising 1732 up-regulated and 1145 down-regulated genes, aligning with Figure 3A. In the comparison between the 30 dph and 15 dph groups, 3536 DEGs were detected, including 1963 up-regulated and 1570 down-regulated genes. Conversely, the number of DEGs identified in the comparison between the 60 dph and 30 dph groups significantly decreased to 1922, consisting of 768 up-regulated and 1154 down-regulated genes. A Venn diagram depicted the overlap of 152 DEGs across the three comparison groups (Figure 3D, Table S6). Cluster analysis based on expression levels revealed diverse expression patterns among these genes. These persistently dynamic genes, which exhibited either up- or down-regulation at specific time points, are likely to play stage-specific biological roles during the development of gill rakers. Among them, 66 genes were also present in the 1839 overlapping DEGs identified in Figure 3B (Figure S1). These genes exhibited high expression levels during specific stages (Figure S2) and are primarily associated with key biological processes, including cytoskeleton organization, immune response, and glycolipid biosynthesis (Table S7).

### 3.4. GO Enrichment Analysis of DEGs

**Figure 4:**
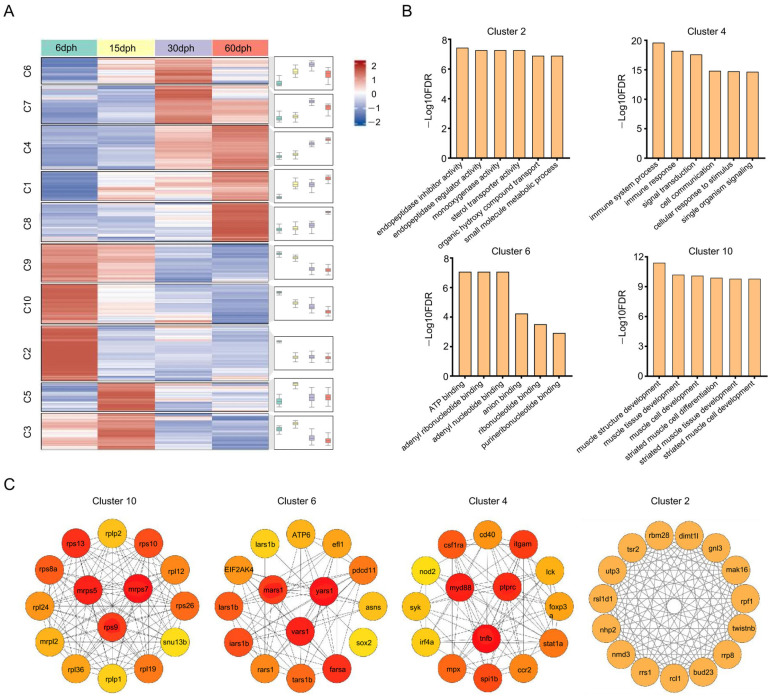
The analysis of DEGs through clustering, GO enrichments, and co-expression networks. (**A**) K-means clustering of DEGs across various developmental stages, with the sizes of each cluster displayed on the left. (**B**) The top five enriched GO terms within the Molecular Function category are identified for Clusters 2, 4, 6, and 10. (**C**) Co-expression networks of the top 15 hub genes in Clusters 2, 4, 6, and 10. The three genes with the highest MCC scores are highlighted in red and positioned centrally within each network.

Following the elimination of duplicate entries, a total of 10,184 DEGs from five comparative groups were aggregated for expression trend analysis. These DEGs were categorized into 10 clusters using K-means clustering (Figure 4A, Table S8). The genes within Cluster 2, characterized by high expression exclusively at 6 dph, Cluster 4, exhibiting continuous upregulation, Cluster 6, displaying a transient peak at 30 dph, and Cluster 10, showing continuous downregulation, were subsequently subjected to GO enrichment analysis (Table S9), with the top five GO terms pertaining to the Molecular Functions (MF) category illustrated in Figure 4B. Genes in Cluster 2 were highly expressed at 6 dph before reverting to baseline, and were significantly enriched in MF terms including small molecule metabolic processes and endopeptidase inhibitor activity. In contrast, Cluster 4 genes showed sustained upregulation during development and were enriched in MF terms related to stimulus response and immune system processes. Cluster 6 genes displayed a transient increase in expression and were predominantly enriched in ATP and purine nucleotide binding activities. Conversely, Cluster 10 genes were progressively downregulated and enriched in MF terms such as muscle structure development and striated muscle cell differentiation. Gene co-expression networks were constructed for representative clusters to identify hub genes based on Maximal Clique Centrality (MCC) (Figure 4C). Genes exhibiting the highest MCC scores were designated as hubs, which included mrps5, mrps7, and rps9 in Cluster 10; mars1, yars1, and vars1 in Cluster 6; and myd88, ptprc, and tnfb in Cluster 4. Conversely, no genes in Cluster 2 satisfied the criteria for hub designation.

### 3.5. KEGG Enrichment Analysis of DEGs

**Figure 5:**
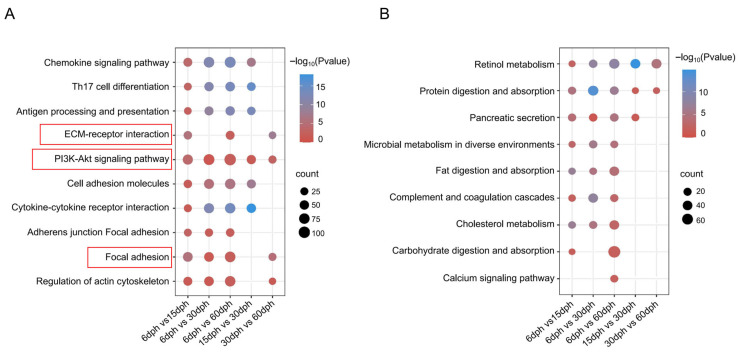
KEGG pathway enrichment during gill raker development in silver carp. (**A**) Bubble diagram displaying significantly enriched KEGG pathways for up-regulated DEGs. (**B**) Bubble diagram showing significantly enriched KEGG pathways for down-regulated DEGs.

To investigate pathway dynamics during development, we stratified the DEGs from each comparison group in Figure 3 into up- and down-regulated sets and performed KEGG enrichment analysis (Tables S10 and S11). Several key pathways were significantly enriched, including immune-related pathways such as the “Cytokine-cytokine receptor interaction” and “Th17 cell differentiation”; pathways involved in signal transduction such as “ECM-receptor interaction”, “Chemokine signaling pathway” and “PI3K-Akt signaling pathway”; and the pathways associated with the formation of cellular community encompass “Focal adhesion”, “Focal adhesion and Adherens junction”, and “Cell adhesion molecules”. The pathway “Regulation of actin cytoskeleton” which governs alterations in cell morphology, migration, proliferation, and survival, was found to be significantly enriched (Figure 5A). Within the down-regulated gene set, a significant proportion of DEGs were predominantly enriched in pathways related to the digestive system, such as protein digestion and absorption, cholesterol metabolism, and lipid metabolism, as well as in metabolic processes including retinol metabolism and microbial metabolism in diverse environments (Figure 5B). Additionally, a KEGG enrichment analysis was conducted on the DEGs common to both comparison methods, comprising 1839 shared genes as depicted in Figure 3B and 152 shared genes as shown in Figure 3D. The analysis revealed that the significantly enriched pathways were largely consistent with those illustrated in Figure 5, encompassing pathways such as “Focal adhesion”, “ECM-receptor interaction”, and the “PI3K-Akt signaling pathway” (Table S12).

### 3.6. GSEA of Focal Adhesion, ECM-Receptor Interaction, and PI3K-Akt Signaling Pathway

**Figure 6:**
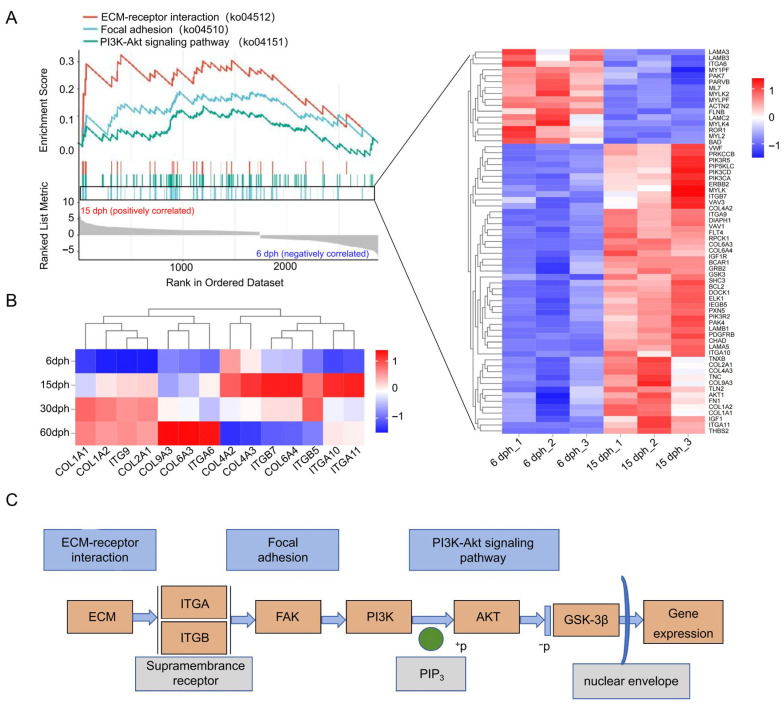
GSEA of key signaling pathways during gill raker development. (**A**) GSEA of the Focal adhesion, ECM-receptor interaction, and PI3K-Akt signaling pathways. The heatmap displays the expression patterns of DEGs in the Focal adhesion pathway between 6 and 15 dph. (**B**) Expression trends of DEGs from the collagen (COL) and integrin (ITG) gene families from 6 to 60 dph. (**C**) A regulatory network of overlapping DEGs across the three KEGG pathways, illustrating their functional interactions.

The pathways “Focal adhesion”, “ECM-receptor interaction”, and “PI3K-Akt signaling” were identified as prominent candidates for further investigation due to their strong statistical significance and relevant biological functions in development. They demonstrated significant enrichment from 6 to 15 dph, with sustained upregulation through 60 dph (Figure 5A), indicating a critical role in gill raker development. We therefore conducted a GSEA focusing on these three pathways in the 6 dph vs. 15 dph comparison (Table S13), where they showed the most pronounced enrichment. The GSEA enrichment plots demonstrated a significant positive correlation of these pathways with the 15 dph group (Figure 6A). This result suggests a coordinated up-regulation of most genes and a pronounced activation of these pathways, driven by the developmental progression. Notably, the Focal adhesion pathway exhibited the highest proportion of up-regulated DEGs. A heatmap was constructed to illustrate the expression patterns of these DEGs between 6 and 15 dph (Figure 6A). Within this pathway, several DEGs were identified as members of the collagen (COL) and integrin (ITG) gene families, implying their potential roles as core regulatory genes. Further analysis of the expression trends of these genes throughout development demonstrated that most were up-regulated relative to their expression at 6 dph. Interestingly, some genes reverted to lower expression levels by 60 dph, which may be associated with the completion of gill raker development at this stage (Figure 6B). These results underscore the critical role of these genes in the gill development of silver carp.

In conclusion, a regulatory network was developed based on the overlapping differentially expressed genes (DEGs) across the three KEGG pathways to elucidate their functional interactions (Figure 6C and Figure S3). Within the extracellular matrix (ECM), collagen (COL) functions as a ligand that binds to its specific receptor, integrin (ITG), located on the cell membrane. This interaction initiates the assembly of focal adhesion complexes on the intracellular side of the membrane, establishing the conditions necessary for the activation of Focal Adhesion Kinase (FAK). Upon autophosphorylation, FAK activates Phosphoinositide 3-kinase (PI3K), which subsequently phosphorylates its substrate PIP2 to produce PIP3. AKT, which contains a PIP3-binding domain, is then recruited and activated. Once activated, AKT transmits the signal downstream to Glycogen Synthase Kinase-3β (GSK-3β). The phosphorylation of GSK-3β by AKT inhibits its kinase activity, thereby preventing the degradation of various growth-related proteins. Additionally, the signal facilitates the translocation of key regulatory factors into the nucleus through the nuclear envelope, ultimately promoting gene expression, facilitating cell cycle progression, and enhancing protein synthesis.

### 3.7. Valadation of RNA-Seq by Quantitative Real-Time PCR

**Figure 7:**
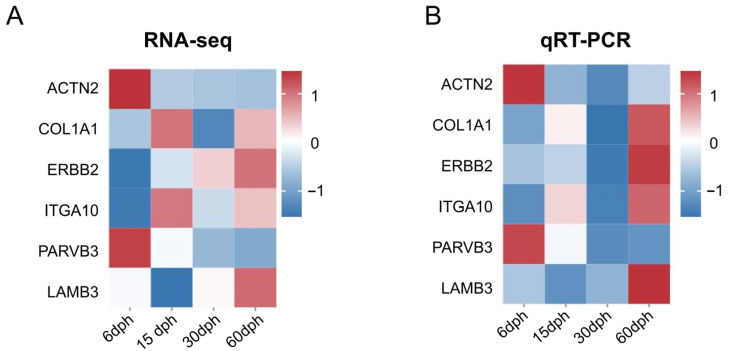
Heatmap illustrating the expression trends of genes in RNA-seq (**A**) and qRT-PCR (**B**). Red indicates up-regulated gene expression, while blue represents down-regulated gene expression.

To validate the reliability of the RNA-seq data, six DEGs were randomly selected for qRT-PCR analysis, including ACTN2 (actinin alpha 2), COL1A1 (collagen type I alpha 1 chain), ERBB2 (erb-b2 receptor tyrosine kinase 2), ITGA10 (integrin subunit alpha 10), PARVB (parvin beta) and LAMB3 (laminin subunit beta 3). The expression trends of these genes, as determined by qRT-PCR, showed a consistent trend with those obtained from RNA-seq (Figure 7); meanwhile, correlation analysis was conducted on RNA-seq and qPCR data, and the results showed a strong positive correlation (R^2^ > 0.8) (Figure S4). Thereby confirming the robustness of the transcriptomic data.

## 4. Discussion

The morphologically specialized gill rakers function as a crucial feeding apparatus in silver carp [36,37]. Their development is tightly coupled with a dietary transition from zooplankton ingestion to phytoplankton filter-feeding [38]. During this developmental trajectory, the gill raker structure evolves from an initially sparse, rod-like configuration to a complex, reticulated architecture [39]. Nevertheless, the molecular mechanisms driving the development of gill rakers remain insufficiently understood. To address the identified research gap and establish a correlation between developmental morphology and gene expression changes, we systematically examined gill raker morphology and performed transcriptome sequencing. The findings revealed that within the body length range of 7.74–16.58 mm, gill raker morphology exhibited distinct variation among groups (Figure 1A–C), and transcriptome samples demonstrated clear separation (Figure 2B). Conversely, at body lengths of 28.94–48.43 mm, the reticulated gill raker structure was largely established and had stabilized (Figure 1D,E). Principal component analysis of the transcriptome further indicated highly consistent gene expression among samples at these later stages (Figure 2B). This finding provides a significant contribution to existing research, suggesting that the gill raker structure is essentially fully developed by the time body length reaches less than 28.94 mm or potentially even earlier.

In order to identify key factors regulating gill raker development in silver carp, we performed differential gene expression analysis across different groups (Figure 3). Two different DEG identification strategies were adopted to capture different aspects of developmental dynamics: sequential comparison (e.g., 66 vs. 15 dph, 15 vs. 30 dph) aimed to identify genes responsible for specific stage transitions, while unified comparison (all stages vs. 6 dph) revealed genes that continuously expressed changes throughout the development process. A total of 10,184 DEGs were identified from five comparative groups. GO enrichment analysis showed that DEGs in Cluster 2 showed high expression at 6 dph before returning to baseline, with significant enrichment in small molecule metabolism and endopeptidase inhibitor activity, indicating early metabolic and enzymatic roles [40]; DEGs in Cluster 6 had a temporary expression spike and were mainly linked to ATP and purine nucleotide binding, emphasizing their involvement in energy metabolism; [41,42] DEGs in Cluster 10 were gradually downregulated, enriched in muscle structure development and striated muscle cell differentiation, indicating a diminishing role in myocyte development as gill rakers mature [43]. Notably, the consistently up-regulated DEGs in cluster 4 were significantly enriched in GO terms related to immune response, signal transduction, and cellular community (Figure 4B), which aligns with the enrichment results from KEGG analysis of up-regulated genes (Figure 5A). This finding reveals that immune maturation is critical during the larval rapid-growth phase [44], while pathways governing cellular communication, growth, and migration are activated in parallel—together pointing to active and vigorous remodeling of the gill raker structure at this stage [45]. Within the down-regulated gene set, a significant proportion of DEGs exhibited substantial enrichment in pathways related to the digestive system (Figure 5B). This coordinated downregulation aligns with the dietary transition in larvae from initial ingestion to filter feeding, indicating a decreased dependence on specific digestive and metabolic processes as the digestive system matures and the diet shifts towards plankton-based food sources [46]. Among these pathways, “Focal adhesion”, “ECM-receptor interaction”, and “PI3K-Akt signaling” remained significant throughout the developmental stages. Notably, within the “Focal adhesion” pathway, many DEGs from the collagen and integrin gene families showed substantial upregulation, underscoring their pivotal roles in focal adhesion formation and gill raker development (Figure 6).

Collagen (COL) was originally identified as a group of proteins characterized by distinct molecular structures, whose fibrous formations contribute to the extracellular scaffold [47]. As the principal structural element of all connective tissues, collagen is also present in the interstitial tissues of nearly all parenchymal organs, where it contributes to the stability and structural integrity of tissues and organs [48]. In vertebrates, collagens (especially type I, II, and X) are well-established markers and essential components of skeletal development, with type I collagen being the primary matrix of bone and type II collagen defining cartilage [49,50]. In a study on *Sparus aurata*, it was observed that gill cells did not express osteoblast markers during early developmental stages. However, expression of COLⅡ—a typical chondrocyte marker—emerged over time, persisted until later stages (60 dph), and then ceased, while strong expression of COLⅠ, recognized as an osteoblast marker, was detected [51]. Based on these findings, we hypothesize that during early gill development in silver carp, chondrocytes may progressively develop toward the osteoblast stage while maintaining high expression levels, though it remains unclear whether chondrocytes directly differentiate into osteoblasts or are simply replaced by them.

Integrins (ITG) are critical cell surface receptors that mediate both cell–cell and cell–extracellular matrix (ECM) adhesion. By binding to ECM ligands, integrins activate specific signal transduction pathways that elicit cellular responses [52]. In bone and cartilage biology, integrin-mediated adhesion (e.g., via α2β1, α10β1, α11β1 binding to collagen) is a fundamental mechanism for osteoblast and chondrocyte attachment to the matrix, directly regulating their survival, function, and tissue remodeling [53]. In our study, at least six integrin subunits (α6, α9, α10, α11, β5, β7) were detected in the gill tissues of silver carp, indicating that integrins represent a major family of cell adhesion molecules essential for cell migration and proliferation [54]. Experiments on *Acanthopagrus schlegelii* have confirmed that abundant integrin expression plays an important role in gill cell migration and gill remodeling [55]. Furthermore, it has been reported that α10 and α11 can combine with β1 to form α10β1 and α11β1 heterodimers, which bind to collagen with a certain preference for collagenⅠ or collagenⅣ [56]. This suggests that the collagen and integrin gene families cooperate to jointly promote gill development in silver carp.

ECM molecules to intracellular cytoskeletal proteins, forming focal adhesions [57]. The FAK signaling protein acts as a molecular switch by binding to these adhesions, thereby activating downstream phosphoinositide 3-kinase (PI3K) [58,59]. Subsequently, PI3K phosphorylates PIP2 to generate the second messenger PIP3, which accumulates at the plasma membrane and further recruits and activates AKT [60,61]. This process is consistent with the observed upregulation of the PI3K-AKT pathway in our experimental results. In summary, we propose a coordinated model for gill development in silver carp. The COL gene family provides the structural scaffold. Concurrently, the ITG family promotes gill cell migration. The PI3K-AKT signaling pathway then regulates the functional maturation and adaptability of these cells. Together, these processes drive gill raker morphogenesis.

## 5. Conclusions

This study provides the first comprehensive transcriptomic atlas of gill raker development in the silver carp, directly linking dynamic gene expression to the morphogenesis of its specialized filter-feeding apparatus. We identify focal adhesion assembly, mediated through the coordinated upregulation of collagen and integrin gene families and activation of the PI3K-Akt signaling pathway, as a central driver of this process. These findings establish a crucial molecular framework connecting genetic regulation to ecological adaptation in a key filter-feeding fish. Future work should focus on functional validation of the identified hub genes and investigating how environmental factors modulate these core pathways to fine-tune gill raker plasticity and filtration efficiency.

## Figures and Tables

**Table 1:** Total length and body weight of silver carp across developmental stages.

Group	6 dph	15 dph	30 dph	45 dph	60 dph
Total length (mm)	7.74 ± 0.55	11.82 ± 0.92	16.58 ± 2.46	28.94 ± 3.93	48.43 ± 5.29
Body weight (mg)	1.50 ± 0.90	11.03 ± 1.94	84.07 ± 4.39	376 ± 31.63	1026.27 ± 297.35
Feeding styles [16]	Swallowing stage (Zooplankton) [16]		Dietary transition stage (Zooplankton & phytoplankton) [16]		Filter-feeding stage (Phytoplankton) [16]

## Data Availability

The raw RNA-seq sequencing data generated in this study are available at the CNCB GSA database (GSA numbers: CRA033098) All data supporting the findings of this study are available within the article and its Supplementary Materials.

## Supplementary Materials

The following supporting information can be downloaded at: https://www.mdpi.com/article/10.3390/biology14121797/s1, Figure S1: Venn diagram illustrating the overlapping DEGs shared by the two analytical methods; Figure S2: The heatmap representing the expression patterns of the 66 shared DEGs; Figure S3: KEGG significant pathway maps of Focal adhesion, ECM-receptor interaction, and PI3K-Akt signaling pathway; Figure S4: Linear regression analysis of the correlation between RNA-seq and RT-qPCR data; Table S1: Primers used for RT-qPCR; Table S2: Transcriptome sequencing data from the gill rackers of silver carp; Table S3: The statistical analysis of gene expression in each sample; Table S4: Differentially expressed genes identified from pairwise comparisons; Table S5: The distribution of differentially expressed genes in Venn diagram of Figure 3B; Table S6: The distribution of differentially expressed genes in Venn diagram of Figure 3D; Table S7: The KEGG pathway enrichment of the 66 shared DEGs; Table S8: DEGs 10 Clusters; Table S9: GO enrichment analysis of differentially expressed genes; Table S10: KEGG enrichment analysis of upregulated differentially expressed genes; Table S11: KEGG enrichment analysis of downregulated differentially expressed genes; Table S12: KEGG enrichment analysis of shared genes; Table S13: GSEA results of the 6 dph vs. 15 dph group.

## Abbreviations

The following abbreviations are used in this manuscript:

dph	days post-hatching
DEGs	Differentially expressed genes
SEM	Scanning electron microscopy
GR	Gill raker
GF	Gill filament
GA	Gill arch
GSEA	Gene Set Enrichment Analysis
COL	Collagen
ITG	Integrins
ECM	extracellular matrix
PI3K	phosphoinositide 3-kinase
CNCB	China National Center for Bioinformation
GSA	Genome Sequence Archive

## References

[1] Hochstrasser J.M., Collins S.F. (2024). Assessing the direct and indirect effects of bigheaded carp (*Hypophthalmichthys* spp.) on freshwater food webs: A meta-analysis. Freshw. Biol..

[2] Hamann L., Schreiber K., Hagenmeyer J., Eduardo S., Spanke T., Blanke A. (2023). Diversity of filter feeding and variations in cross-flow filtration of five ram-feeding fish species. Front. Mar. Sci..

[3] Cohen K.E., Hernandez L.P. (2018). Making a master filterer: Ontogeny of specialized filtering plates in silver carp (*Hypophthalmichthys molitrix*). J. Morphol..

[4] Cheer A., Cheung S., Hung T.-C., Piedrahita R.H., Sanderson S.L. (2011). Computational Fluid Dynamics of Fish Gill Rakers During Crossflow Filtration. Bull. Math. Biol..

[5] Sanderson S.L., Cheer A.Y., Goodrich J.S., Graziano J.D., Callan W.T. (2001). Crossflow filtration in suspension-feeding fishes. Nature.

[6] Cangelosi A.A., Mays N.L., Balcer M.D., Reavie E.D., Reid D.M., Sturtevant R., Gao X. (2007). The response of zooplankton and phytoplankton from the North American Great Lakes to filtration. Harmful Algae.

[7] Conover R.J., Mayzaud P. (1984). Utilization of Phytoplankton by Zooplankton during the Spring Bloom in a Nova Scotia Inlet. Can. J. Fish. Aquat. Sci..

[8] Kahilainen K.K., Siwertsson A., Gjelland K.Ø., Knudsen R., Bøhn T., Amundsen P.-A. (2010). The role of gill raker number variability in adaptive radiation of coregonid fish. Evol. Ecol..

[9] Amundsen P.A., Bøhn T., Vaga G.H. (2004). Gill raker morphology and feeding ecology of two sympatric morphs of European whitefish (*Coregonus lavaretus*). Ann. Zool. Fenn..

[10] Xie S., Cui Y., Li Z. (2001). Dietary-morphological relationships of fishes in Liangzi Lake, China. J. Fish Biol..

[11] Häkli K., Østbye K., Kahilainen K.K., Amundsen P., Præbel K. (2018). Diversifying selection drives parallel evolution of gill raker number and body size along the speciation continuum of European whitefish. Ecol. Evol..

[12] Zhang Y., Huang D., Li X., Liu Q., Li J., Li Y., Yang J., Zhu S. (2020). Analysis of Fish Community Structure and Environmental Impact in the Xijiang River Southern. Fish. Sci..

[13] Witkop E.M., Van Wassenbergh S., Heideman P.D., Sanderson S.L. (2023). Biomimetic models of fish gill rakers as lateral displacement arrays for particle separation. Bioinspiration Biomim..

[14] Xie P. (2003). Silver Carp and Bighead Carp, and Their Use in the Control of Algal Blooms.

[15] Li X., Li F., Zou G., Feng C., Sha H., Liu S., Liang H. (2021). Physiological responses and molecular strategies in heart of silver carp (*Hypophthalmichthys molitrix*) under hypoxia and reoxygenation. Comp. Biochem. Physiol. Part D Genom. Proteom..

[16] Liu H., Li M., Li L., Zhu W. (1993). Research on the Postembryonic, Development Biology of the Filtering-feeding organs of Silver carp. J. Dalian Fish. Univ..

[17] Battonyai I., Specziár A., Vitál Z., Mozsár A., Görgényi J., Borics G., Tóth L.G., Boros G. (2015). Relationship between gill raker morphology and feeding habits of hybrid bigheaded carps (*Hypophthalmichthys* spp.). Knowl. Manag. Aquat. Ecosyst..

[18] Cohen K.E., George A.E., Chapman D.C., Chick J.H., Hernandez L.P. (2020). Developmental ecomorphology of the epibranchial organ of the silver carp, *Hypophthalmichthys molitrix*. J. Fish Biol..

[19] Li X., Ling C., Wang Q., Feng C., Luo X., Sha H., He G., Zou G., Liang H. (2022). Hypoxia Stress Induces Tissue Damage, Immune Defense, and Oxygen Transport Change in Gill of Silver Carp (*Hypophthalmichthys molitrix*): Evaluation on Hypoxia by Using Transcriptomics. Front. Mar. Sci..

[20] Shang F., Bao M., Liu F., Hu Z., Wang S., Yang X., Yu Y., Zhang H., Jiang C., Qiu X. (2022). Transcriptome profiling of tiger pufferfish (*Takifugu rubripes*) gills in response to acute hypoxia. Aquaculture.

[21] Cheng X., Li F., Lu J., Wen Y., Li Z., Liao J., Cao J., He X., Sun J., Liu Q. (2024). Transcriptome analysis in gill reveals the adaptive mechanism of domesticated common carp to the high temperature in shallow rice paddies. Aquaculture.

[22] Blondeau-Bidet E., Tine M., Gonzalez A.A., Guinand B., Lorin-Nebel C. (2024). Coping with salinity extremes: Gill transcriptome profiling in the black-chinned tilapia (*Sarotherodon melanotheron*). Sci. Total Environ..

[23] Xia Y., Li X., Yang J., Zhu S., Wu Z., Li J., Li Y. (2021). Elevated Temperatures Shorten the Spawning Period of Silver Carp (*Hypophthalmichthys molitrix*) in a Large Subtropical River in China. Front. Mar. Sci..

[24] Zhang Z., Shi Y., Zhang J., Liu Q. (2022). Experimental observation on the effects of bighead carp (*Hypophthalmichthys nobilis*) on the plankton and water quality in ponds. Environ. Sci. Pollut. Res..

[25] Feng N., Li X., Sha H., Luo X., Zou G., Zhang J., Liang H. (2025). A 5′ Promoter Region SNP in CTSC Leads to Increased Hypoxia Tolerance in Changfeng Silver Carp (*Hypophthalmichthys molitrix*). Animals.

[26] Sheng Q., Vickers K., Zhao S., Wang J., Samuels D.C., Koues O., Shyr Y., Guo Y. (2016). Multi-perspective quality control of Illumina RNA sequencing data analysis. Brief. Funct. Genom..

[27] Bush S.J. (2020). Read trimming has minimal effect on bacterial SNP-calling accuracy. Microb. Genom..

[28] Alvarez R.V., Pongor L.S., Mariño-Ramírez L., Landsman D. (2018). TPMCalculator: One-step software to quantify mRNA abundance of genomic features. Bioinformatics.

[29] Liu S., Wang Z., Zhu R., Wang F., Cheng Y., Liu Y. (2021). Three Differential Expression Analysis Methods for RNA Sequencing: Limma, EdgeR, DESeq2. J. Vis. Exp. JoVE.

[30] Van Aken B. (2019). Response to the note to editor: Comments on “transcriptomic response of *Arabidopsis thaliana* exposed to hydroxylated polychlorinated biphenyls (OH-PCBs)”. Int. J. Phytoremediat..

[31] Xu S., Hu E., Cai Y., Xie Z., Luo X., Zhan L., Tang W., Wang Q., Liu B., Wang R. (2024). Using clusterProfiler to characterize multiomics data. Nat. Protoc..

[32] Chen G., Zhou T., Cao J., Zou G., Liang H. (2024). Comparative transcriptome sequencing and weighted coexpression network analysis reveal growth-related hub genes and key pathways in the Chinese soft-shelled turtle (*Pelodiscus sinensis*). Water Biol. Secur..

[33] Van der Auwera G.A., Carneiro M.O., Hartl C., Poplin R., Del Angel G., Levy-Moonshine A., Jordan T., Shakir K., Roazen D., Thibault J. (2013). From FastQ Data to High-Confidence Variant Calls: The Genome Analysis Toolkit Best Practices Pipeline. Curr. Protoc. Bioinform..

[34] Livak K.J., Schmittgen T.D. (2001). Analysis of Relative Gene Expression Data Using Real-Time Quantitative PCR and the 2^−ΔΔCT^ Method. Methods.

[35] Miller M., Pham A.K., Gonen A., Navia-Pelaez J.M., Xia K., Park S., Osterman A.L., Bacon K., Beaton G., Kurten R.C. (2022). Reduced AIBP expression in bronchial epithelial cells of asthmatic patients: Potential therapeutic target. Clin. Exp. Allergy.

[36] Zhou Q., Xie P., Xu J., Ke Z., Guo L. (2009). Growth and food availability of silver and bighead carps: Evidence from stable isotope and gut content analysis. Aquac. Res..

[37] Cremer M.C., Smitherman R.O. (1980). Food habits and growth of silver and bighead carp in cages and ponds. Aquaculture.

[38] Esmaeili H.R., Johal M.S. (2015). Food and feeding habits of silver carp, *Hypophthalmichthys molitrix* (Val. 1844) in Gobindsagar Reservoir, India. Int. J. Aquat. Biol..

[39] Walleser L.R., Howard D.R., Sandheinrich M.B., Gaikowski M.P., Amberg J.J. (2014). Confocal microscopy as a useful approach to describe gill rakers of Asian species of carp and native filter-feeding fishes of the upper Mississippi River system. J. Fish Biol..

[40] Singh M., Xavier J., Tamhankar S.P., Amin R. (2024). Discovery of novel small molecule Asparaginyl endopeptidase inhibitors via dual approach-based virtual screening and molecular simulation studies. Alzheimer’s Dement..

[41] Lopez-Schenk R., Collins N.L., Schenk N.A., Beard D.A. (2023). Integrated Functions of Cardiac Energetics, Mechanics, and Purine Nucleotide Metabolism. Compr. Physiol..

[42] Huang Z., Xie N., Illes P., Di Virgilio F., Ulrich H., Semyanov A., Verkhratsky A., Sperlagh B., Yu S.-G., Huang C. (2021). From purines to purinergic signalling: Molecular functions and human diseases. Signal Transduct. Target. Ther..

[43] Zhan S., Zhai H., Tang M., Xue Y., Li D., Wang L., Zhong T., Dai D., Cao J., Guo J. (2022). Profiling and Functional Analysis of mRNAs during Skeletal Muscle Differentiation in Goats. Animals.

[44] Balseiro P., Moreira R., Chamorro R., Figueras A., Novoa B. (2013). Immune responses during the larval stages of *Mytilus galloprovincialis*: Metamorphosis alters immunocompetence, body shape and behavior. Fish Shellfish. Immunol..

[45] Jonz M.G. (2024). Cell proliferation and regeneration in the gill. J. Comp. Physiol. B-Biochem. Syst. Environ. Physiol..

[46] Bonelli M., Bruno D., Brilli M., Gianfranceschi N., Tian L., Tettamanti G., Caccia S., Casartelli M. (2020). Black Soldier Fly Larvae Adapt to Different Food Substrates through Morphological and Functional Responses of the Midgut. Int. J. Mol. Sci..

[47] Laronha H., Caldeira J. (2020). Structure and Function of Human Matrix Metalloproteinases. Cells.

[48] Gelse K., Pöschl E., Aigner T. (2003). Collagens—Structure, function, and biosynthesis. Adv. Drug Deliv. Rev..

[49] Chen Y., Yang S., Lovisa S., Ambrose C.G., McAndrews K.M., Sugimoto H., Kalluri R. (2021). Type-I collagen produced by distinct fibroblast lineages reveals specific function during embryogenesis and Osteogenesis Imperfecta. Nat. Commun..

[50] Ahmed S., Nowlan N.C. (2020). Initiation and emerging complexity of the collagen network during prenatal skeletal development. Eur. Cells Mater..

[51] Estêvão M.D., Silva N., Redruello B., Costa R., Gregório S., Canário A.V.M., Power D.M. (2011). Cellular morphology and markers of cartilage and bone in the marine teleost Sparus auratus. Cell Tissue Res..

[52] Kanchanawong P., Calderwood D.A. (2022). Organization, dynamics and mechanoregulation of integrin-mediated cell–ECM adhesions. Nat. Rev. Mol. Cell Biol..

[53] Loeser R.F. (2014). Integrins and chondrocyte–matrix interactions in articular cartilage. Matrix Biol..

[54] Hu X., Roy S.R., Jin C., Li G., Zhang Q., Asano N., Asahina S., Kajiwara T., Takahara A., Feng B. (2022). Control cell migration by engineering integrin ligand assembly. Nat. Commun..

[55] Liu Q., Guo L., Zhang H., Ge J., Luo J., Song K., Zhao L., Yang S. (2024). Hypoxia induces reversible gill remodeling in largemouth bass (*Micropterus salmoides*) through integrins-mediated cell adhesion. Fish Shellfish Immunol..

[56] Khoshnoodi J., Pedchenko V., Hudson B.G. (2008). Mammalian collagen IV. Microsc. Res. Tech..

[57] Matrullo G., Filomeni G., Rizza S. (2025). Redox regulation of focal adhesions. Redox Biol..

[58] Schneider G.B., Zaharias R., Seabold D., Stanford C. (2011). Integrin-associated tyrosine kinase FAK affects Cbfa1 expression. J. Orthop. Res..

[59] Parsons J.T. (2003). Focal adhesion kinase: The first ten years. J. Cell Sci..

[60] Engelman J.A., Luo J., Cantley L.C. (2006). The evolution of phosphatidylinositol 3-kinases as regulators of growth and metabolism. Nat. Rev. Genet..

[61] Manning B.D., Cantley L.C. (2007). AKT/PKB Signaling: Navigating Downstream. Cell.

