# Supplementary material for: Comparative Transcriptome Sequencing Analysis Revealed Key Pathways and Hub Genes Related to Gill Raker Development in Silver Carp (Hypophthalmichthys molitrix)
Source: Biology (Basel). 2025 Dec 17;14(12):1797. doi: 10.3390/biology14121797 (PMC12730290; doi:10.3390/biology14121797)

**Figure S1.** Venn diagram illustrating the overlapping differentially expressed genes (DEGs) shared by the two analytical methods.


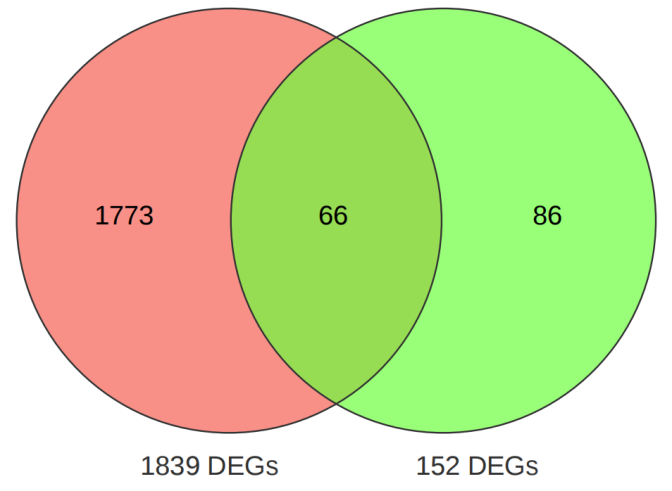

Supplement: Supplementary file 1 [file biology-14-01797-s001.zip › Figure S1.docx]
