# Supplementary material for: Comparative Transcriptome Sequencing Analysis Revealed Key Pathways and Hub Genes Related to Gill Raker Development in Silver Carp (Hypophthalmichthys molitrix)
Source: Biology (Basel). 2025 Dec 17;14(12):1797. doi: 10.3390/biology14121797 (PMC12730290; doi:10.3390/biology14121797)
Supplement: Supplementary file 1 [file biology-14-01797-s001.zip › Figure S3.docx]

**Figure S3. KEGG significant pathway maps of Focal adhesion, ECM-receptor interaction, and PI3K-Akt signaling pathway.**


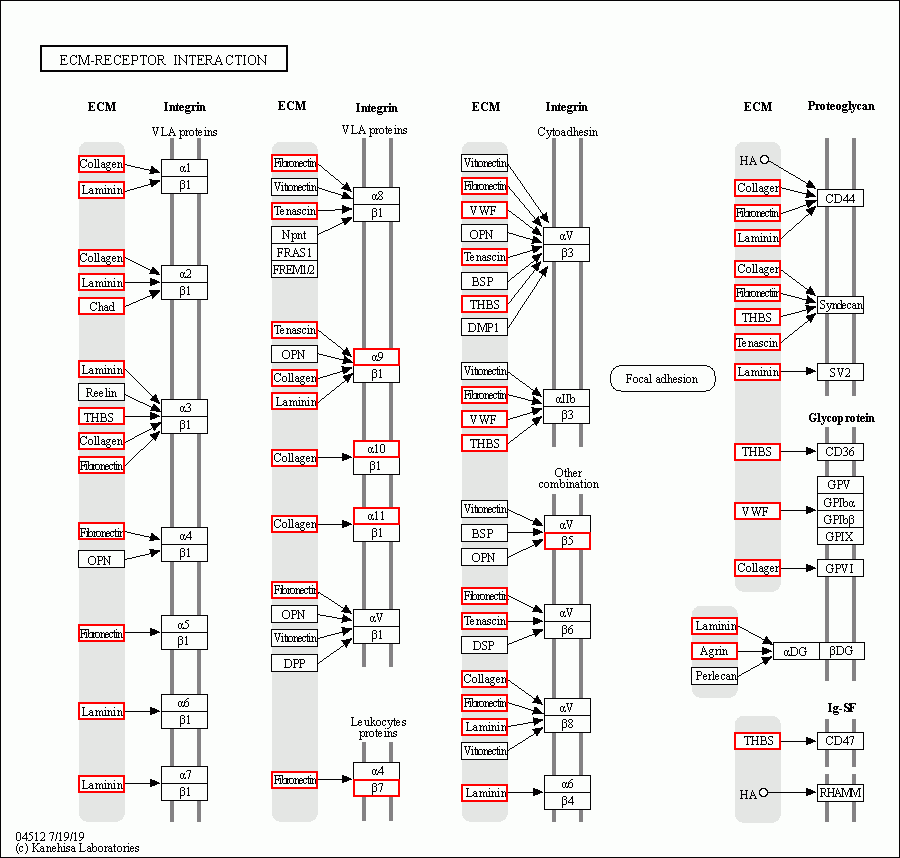

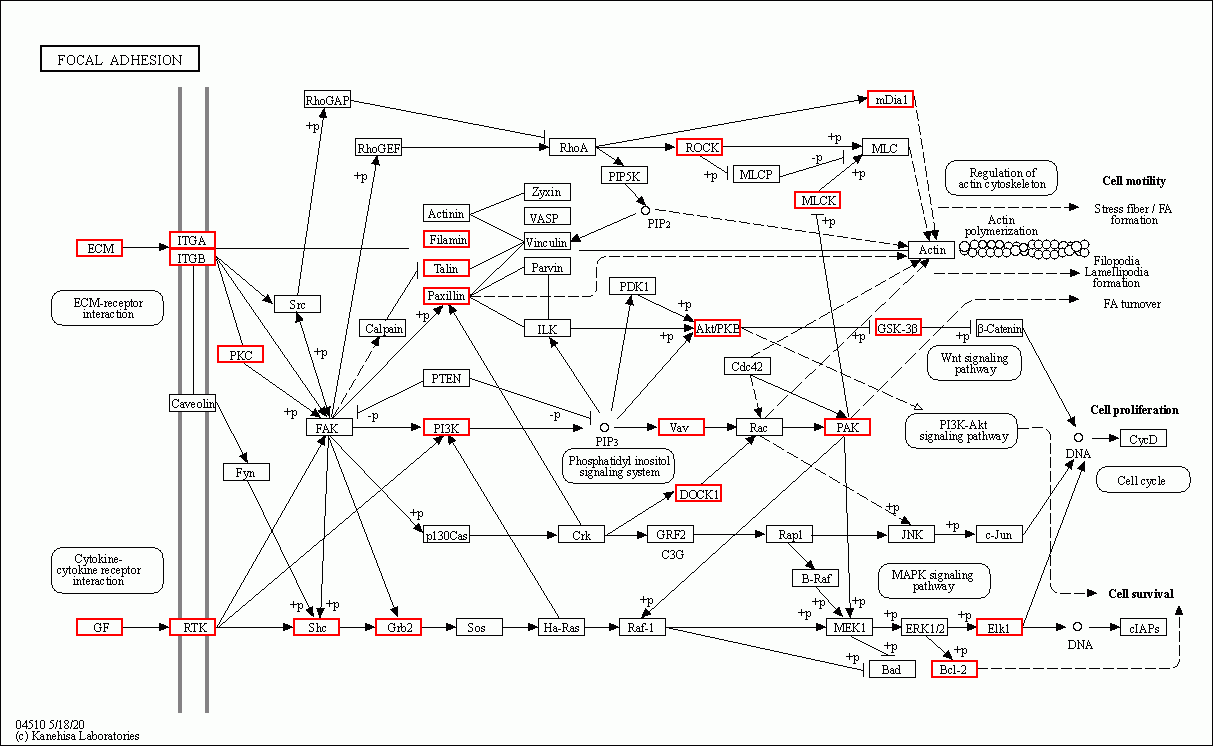

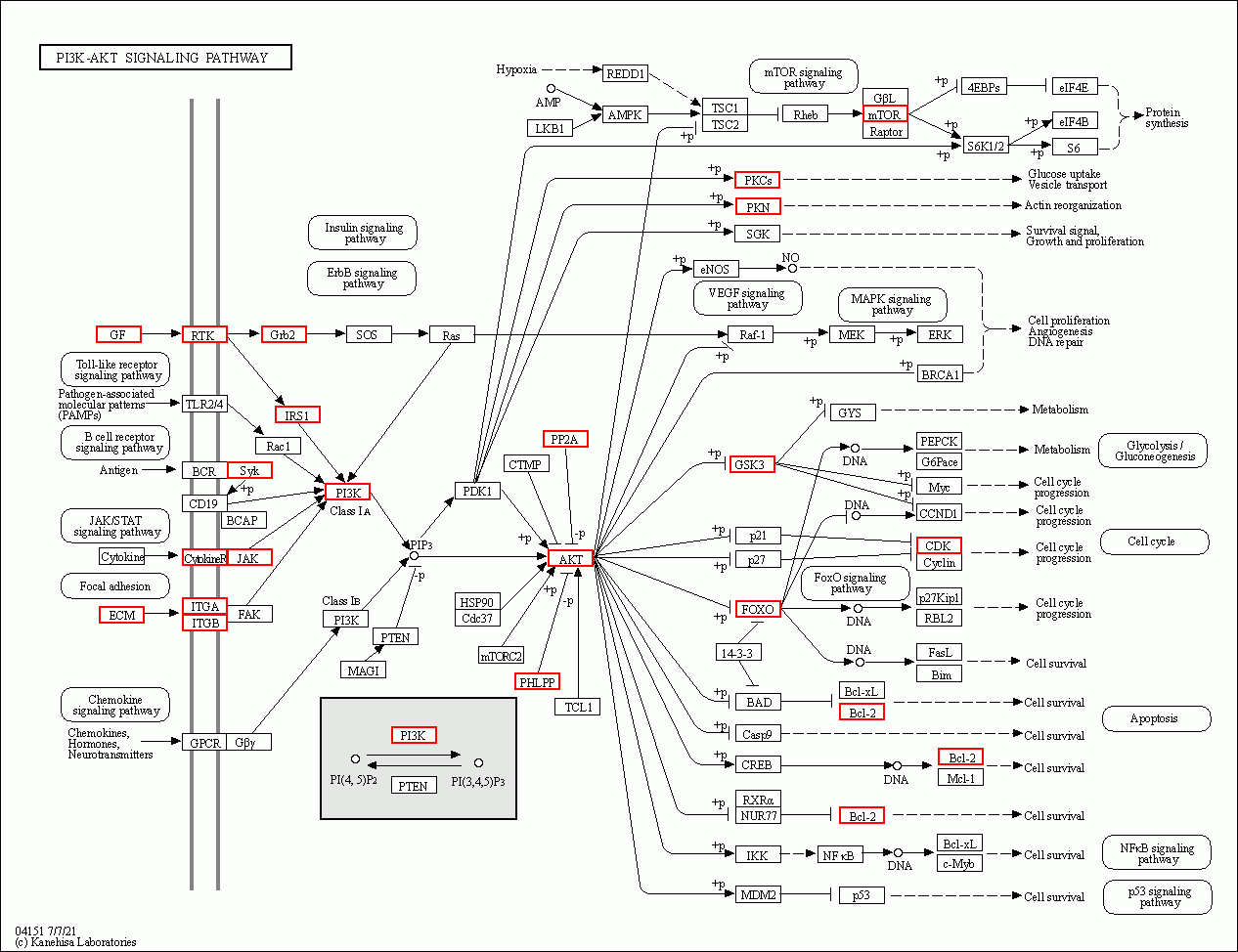


Note: Differentially expressed genes on the pathway are highlighted with red boxes.
