# Supplementary material for: Comparative Transcriptome Sequencing Analysis Revealed Key Pathways and Hub Genes Related to Gill Raker Development in Silver Carp (Hypophthalmichthys molitrix)
Source: Biology (Basel). 2025 Dec 17;14(12):1797. doi: 10.3390/biology14121797 (PMC12730290; doi:10.3390/biology14121797)
Supplement: Supplementary file 1 [file biology-14-01797-s001.zip › Figure S4.docx]

**Figure S4. Linear regression analysis of the correlation between RNA-seq and RT-qPCR data.**

| Comparison groups | Scatter plot of RNA-seq vs RT-qPCR data |
| --- | --- |
| 6 dph vs 15 dph | 6 dph vs 15 dph  qPCR Fold-change  RNA-seq Fold-change |
| 6 dph vs 30 dph | 6 dph vs 30 dph  RNA-seq Fold-change  qPCR Fold-change |
| 6 dph vs 60 dph | RNA-seq Fold-change  6 dph vs 60 dph  qPCR Fold-change |
| 15 dph vs 30 dph | 15 dph vs 30 dph  RNA-seq Fold-change  qPCR Fold-change |
| 30 dph vs 60 dph | 30 dph vs 60 dph  qPCR Fold-change  RNA-seq Fold-change |
