# Supplementary material for: Comparative Transcriptome Sequencing Analysis Revealed Key Pathways and Hub Genes Related to Gill Raker Development in Silver Carp (Hypophthalmichthys molitrix)
Source: Biology (Basel). 2025 Dec 17;14(12):1797. doi: 10.3390/biology14121797 (PMC12730290; doi:10.3390/biology14121797)
Supplement: Supplementary file 1 [file biology-14-01797-s001.zip › Table S1.docx]

**Table S1. Primers used for RT-qPCR.**

| **Primer names** | **Primer sequences (5′–3′)** |
| --- | --- |
| 40s F | GTTTCCGCAAGGCTCAGTGT |
| 40s R | TCTCCTCACGGTTCCAGCAC |
| ACTN2 F | TGAGGGCAAGATGGTGTCGG |
| ACTN2 R | CCTGCTCAACATCTCCTCCTTA |
| COL1A1 F | TGCCCAGACGACGATTTC |
| COL1A1 R | CATTGCTCCGCCAGATTT |
| ERBB2 F | CATCTCACCCACTTTACCCG |
| ERBB2 R | CACTCGTTCACTCACGCACA |
| ITGA10 F | AGCAAGGGAAGGAGCCACTA |
| ITGA10 R | CCCACCATGCCAAACAAG |
| LAMB3 F | ATGGTGGCAGTCCAAGAAAG |
| LAMB3 R | GGCAGCGAGTAGCAGTAAGTG |
| PARVB3 F | GGAGGAGGACCGAATCATCG |
| PARVB3 R | AGCAGGTACACGATGGACAC |
