# Supplementary material for: Comparative Transcriptome Sequencing Analysis Revealed Key Pathways and Hub Genes Related to Gill Raker Development in Silver Carp (Hypophthalmichthys molitrix)
Source: Biology (Basel). 2025 Dec 17;14(12):1797. doi: 10.3390/biology14121797 (PMC12730290; doi:10.3390/biology14121797)
Supplement: Supplementary file 1 [file biology-14-01797-s001.zip › Table S2.docx]

**Table S2. Transcriptome sequencing data from the gill rackers of silver carp.**

| **Group** | **Raw_Reads** | **Clean_Reads** | **Q20 (%)** | **Q30 (%)** | **Total_Mapped** | **Mapped_to_Gene** | **Mapped_to_Inter Gene** | **Mapped_to_Exons** |
| --- | --- | --- | --- | --- | --- | --- | --- | --- |
| 6dph_1 | 50,056,042 | 49,261,216 | 98.04 | 96.26 | 45,282,177 (91.92%) | 38,081,413 (86.71%) | 5,837,032 (13.29%) | 35,960,722 (94.43%) |
| 6dph_2 | 46,673,176 | 45,887,466 | 98.01 | 96.22 | 42,334,540 (92.26%) | 35,293,469 (86.02%) | 5,737,945 (13.98%) | 33,131,868 (93.88%) |
| 6dph_3 | 48,131,522 | 47,389,962 | 98.09 | 96.35 | 43,841,672 (92.51%) | 37,220,969 (87.68%) | 5,231,456 (12.32%) | 35,415,181 (95.15%) |
| 15dph_1 | 49,77,1662 | 48,992,040 | 98.12 | 96.39 | 45,587,921 (93.05%) | 39,327,098 (89.33%) | 4,698,072 (10.67%) | 37,695,379 (95.85%) |
| 15dph_2 | 45,851,774 | 45,175,040 | 98.13 | 96.42 | 41,833,170 (92.60%) | 35,962,781 (88.98%) | 4,452,954 (11.02%) | 34,380,510 (95.60%) |
| 15dph_3 | 45,555,656 | 44,879,692 | 98.11 | 96.37 | 41,471,174 (92.41%) | 35,797,812 (89.22%) | 4,324,907 (10.78%) | 33,929,131 (94.78%) |
| 30dph_1 | 48,642,880 | 47,870,274 | 98.04 | 96.23 | 43,568,697 (91.01%) | 37,386,139 (88.64%) | 4,792,883 (11.36%) | 35,361,134 (94.58%) |
| 30dph_2 | 47,741,520 | 46,985,000 | 98.04 | 96.25 | 42,619,492 (90.71%) | 36,473,512 (88.36%) | 4,806,416 (11.64%) | 34,254,153 (93.92%) |
| 30dph_3 | 50,346,664 | 49,536,272 | 98.10 | 96.36 | 45,191,885 (91.23%) | 38,868,138 (88.91%) | 4,847,520 (11.09%) | 36,931,089 (95.02%) |
| 45dph_1 | 58,755,476 | 57,849,784 | 98.12 | 96.42 | 53,159,897 (91.89%) | 45,685,539 (88.67%) | 5,837,612 (11.33%) | 43,268,665 (94.71%) |
| 45dph_2 | 56,581,770 | 55,710,222 | 98.09 | 96.34 | 51,187,896 (91.88%) | 44,253,216 (89.27%) | 5,316,583 (10.73%) | 41,945,984 (94.79%) |
| 45dph_3 | 60,832,704 | 59,828,138 | 98.08 | 96.31 | 54,835,871 (91.66%) | 47,268,794 (88.88%) | 5,915,341 (11.12%) | 44,741,978 (94.65%) |
| 60dph_1 | 49,061,136 | 48,203,908 | 97.85 | 95.95 | 44,573,968 (92.47%) | 38,020,525 (87.88%) | 5,245,875 (12.12%) | 35,749,902 (94.03%) |
| 60dph_2 | 51,879,790 | 50,925,034 | 97.81 | 95.89 | 46,886,787 (92.07%) | 40,288,426 (88.56%) | 5,205,303 (11.44%) | 38,108,170 (94.59%) |
| 60dph_3 | 51,886,296 | 51,107,086 | 97.95 | 96.14 | 47,389,191 (92.73%) | 40,365,725 (87.92%) | 5,546,060 (12.08%) | 38,214,499 (94.67%) |
