# Supplementary figures and images for: Comparative Transcriptome Sequencing Analysis Revealed Key Pathways and Hub Genes Related to Gill Raker Development in Silver Carp (Hypophthalmichthys molitrix)
Source: Biology (Basel). 2025 Dec 17;14(12):1797. doi: 10.3390/biology14121797 (PMC12730290; doi:10.3390/biology14121797)

**Figure S2. The heatmap representing the expression patterns of the 66 shared DEGs.**

**
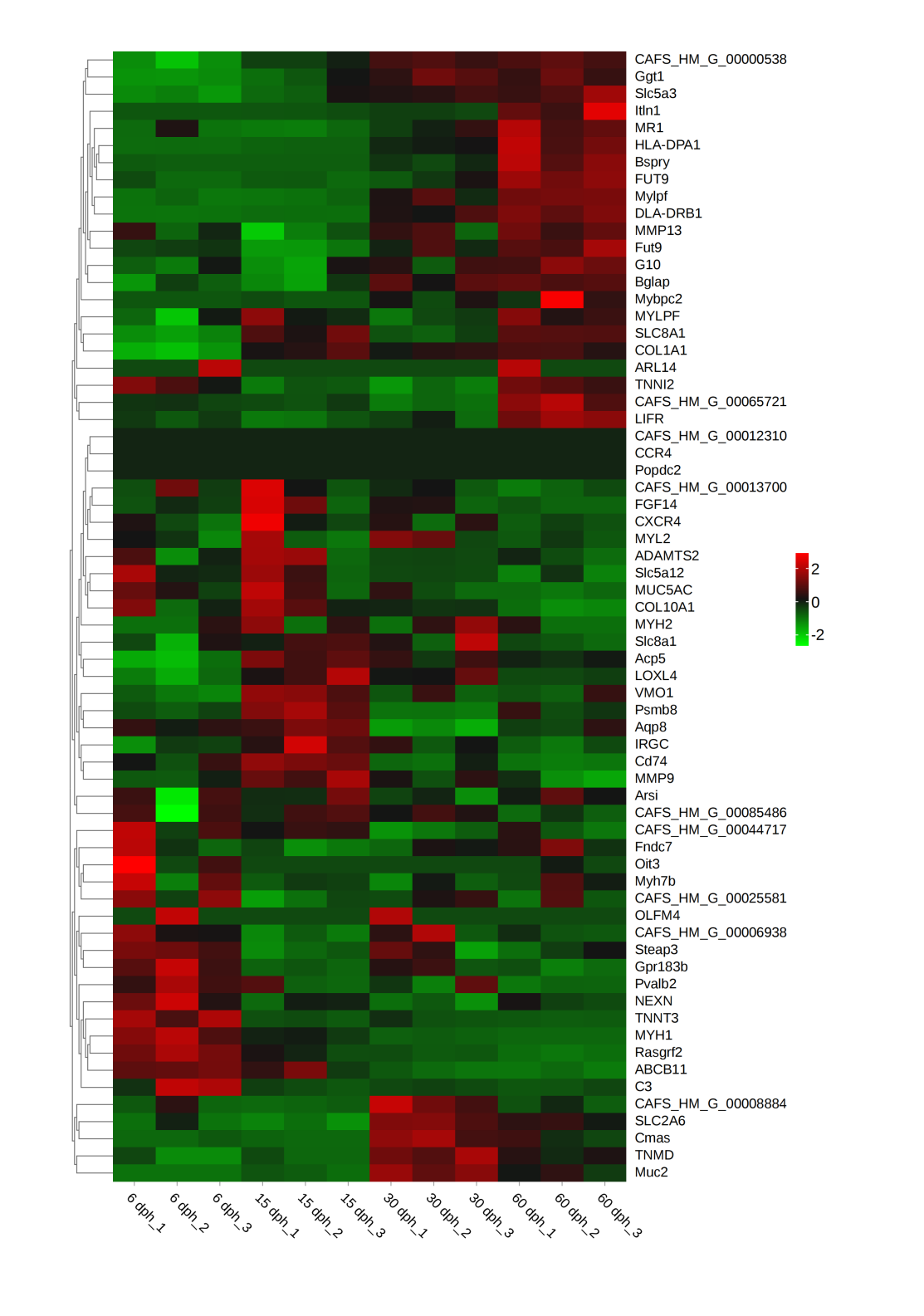
**

Supplement: Supplementary file 1 [file biology-14-01797-s001.zip › Figure S2.docx]
